# Mitochondrial impairment and repair in the pathogenesis of systemic lupus erythematosus

**DOI:** 10.3389/fimmu.2022.929520

**Published:** 2022-07-25

**Authors:** Like Zhao, Xianda Hu, Fei Xiao, Xuan Zhang, Lidan Zhao, Min Wang

**Affiliations:** ^1^ Department of Rheumatology, Beijing Hospital, National Center of Gerontology, Institute of Geriatric Medicine, Clinical Immunology Center, Chinese Academy of Medical Sciences and Peking Union Medical College, Beijing, China; ^2^ Beijing Tibetan Hospital, China Tibetology Research Center, Beijing, China; ^3^ The Key Laboratory of Geriatrics, Beijing Institute of Geriatrics, Beijing Hospital, National Center of Gerontology, National Health Commission, Institute of Geriatric Medicine, Chinese Academy of Medical Sciences, Beijing, China; ^4^ Clinical Biobank, Beijing Hospital, National Center of Gerontology, Institute of Geriatric Medicine, Chinese Academy of Medical Sciences, Beijing, China; ^5^ Department of Rheumatology and Clinical Immunology, Peking Union Medical College Hospital, Chinese Academy of Medical Sciences and Peking Union Medical College, Beijing, China; ^6^ National Clinical Research Center for Dermatologic and Immunologic Diseases (NCRC-DID), Ministry of Science and Technology, Beijing, China

**Keywords:** systemic lupus erythematosus, mitochondrial reactive oxygen species, mitochondrial dysfunction, mitophagy, oxidative stress, mitochondria targeting therapeutics

## Abstract

Nucleic acid autoantibodies, increase type I interferon (IFN-α) levels, and immune cell hyperactivation are hallmarks of systemic lupus erythematosus (SLE). Notably, immune cell activation requires high level of cellular energy that is predominately generated by the mitochondria. Mitochondrial reactive oxygen species (mROS), the byproduct of mitochondrial energy generation, serves as an essential mediator to control the activation and differentiation of cells and regulate the antigenicity of oxidized nucleoids within the mitochondria. Recently, clinical trials on normalization of mitochondrial redox imbalance by mROS scavengers and those investigating the recovery of defective mitophagy have provided novel insights into SLE prophylaxis and therapy. However, the precise mechanism underlying the role of oxidative stress-related mitochondrial molecules in skewing the cell fate at the molecular level remains unclear. This review outlines distinctive mitochondrial functions and pathways that are involved in immune responses and systematically delineates how mitochondrial dysfunction contributes to SLE pathogenesis. In addition, we provide a comprehensive overview of damaged mitochondrial function and impaired metabolic pathways in adaptive and innate immune cells and lupus-induced organ tissues. Furthermore, we summarize the potential of current mitochondria-targeting drugs for SLE treatment. Developing novel therapeutic approaches to regulate mitochondrial oxidative stress is a promising endeavor in the search for effective treatments for systemic autoimmune diseases, particularly SLE.

## Introduction

Systemic lupus erythematosus (SLE) is a chronic autoimmune disease characterized by self-tolerance breakdown, excessive production of autoantibodies and impaired clearance of immune complexes, which incites sustained inflammatory response and multi-organ damage (1). As the signature of SLE, excessive production of type I IFN (especially IFN-α) substantially contributes to SLE pathogenesis by triggering the activation and expansion of autoreactive immune cells, promoting autoantibodies production and inflammatory cytokines release, hence leading to the formation of nucleic acid-protein immune complex and perpetuating the autoreactive immune response. Despite great improvement has been achieved in SLE survival, the morbidity from both the disease and the medications makes the prognosis still far from satisfactory (2). Therefore, deeper understanding and further elucidation of the cellular and molecular immune aberrations in SLE pathogenesis are still in desire.

Cellular bioactivity cannot be accomplished without energy and mitochondria is the critical organelle in initiating and maintaining cellular energy metabolism. By generating adenosine triphosphate (ATP) through oxidative phosphorylation, mitochondria provide energy for multiple cell activities and determine the cell fate like activation and differentiation (3). In addition, mitochondria are also the major producer of reactive oxygen species (ROS), which are involved in redox balance and oxidative damage, and regulate iron homeostasis, calcium efflux, and metabolic pathways (4). Abnormal mitochondrial function participate in numerous diseases, such as neurological disorders, endocrinopathy, inflammatory bowel disease, and autoimmune diseases (5). Moreover, under oxidative stress conditions, immune complexes and IFN-α stimulate excessive mitochondrial ROS (mROS) production that consequently induces highly immunogenic oxidative mitochondrial DNA (Ox-mtDNA). This Ox-mtDNA activates inflammatory signaling pathways and promotes cell death progression (6, 7). Notably, several studies have reported that mitochondrial dysfunction is closely associated with SLE (8, 9). Furthermore, regulation of excessive levels of mROS and alleviation of deleterious consequences of mitochondrial dysfunction have emerged as promising therapeutic approaches for SLE treatment (9, 10).

This review aimed to summarize the current research advances on physiological and pathological roles of mitochondria., Mitophagy defects of antioxidant defense and mitochondrial dysfunction contributing to SLE immunopathogenesis were systematically reviewed. The drugs based on the normalization of mitochondria function and mitophagy metabolic pathways were briefly introduced and their potential therapeutic values in SLE were discussed.

## Mitochondrial functions and homeostasis maintenance

### Mitochondrial functions

Mitochondria are energy-producing cellular organelles that generate energy in the form of ATP *via* oxidative phosphorylation (OXPHOS) performed by the electron transport chain (ETC) complexes. These complexes consist of five enzymes, wherein complexes I and III are the primary enzymes that catalyze the generation of low concentrations of superoxide anions. These enzymes maintain the mitochondrial antioxidant defenses (11). However, increased mROS levels during oxidative stress cause mitochondrial DNA (mtDNA) impairment and ETC defects, resulting in mitochondrial dysfunction (12).

**Figure 1:**
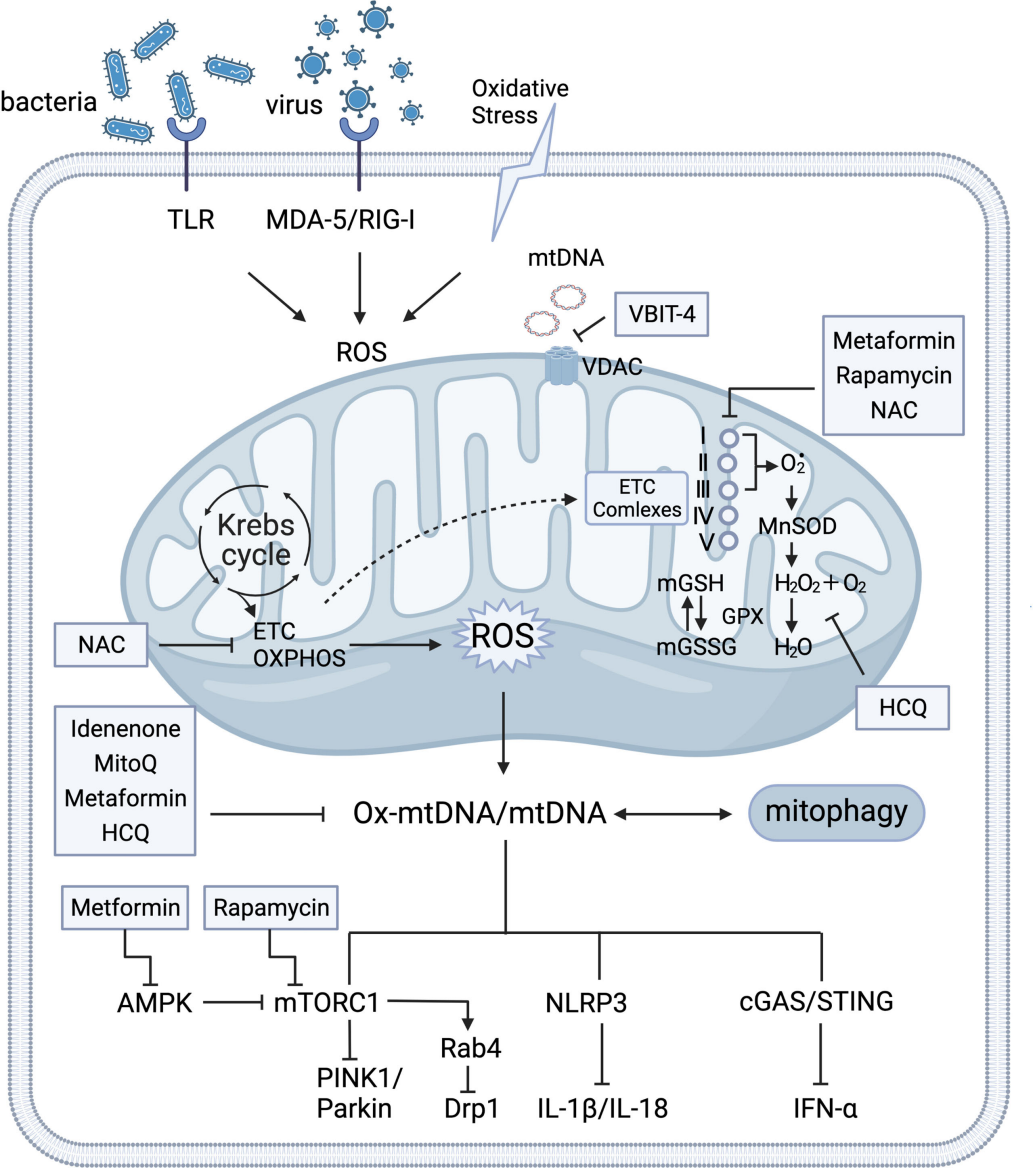
Crosstalk of mitochondria homeostasis and immune signaling. Mitochondria support the major generation of reactive oxygen species(mROS) for modulating various signaling and cellular metabolism. Mitochondria produce ATP through the tricarboxylic acid cycle (TCA) oxidative phosphorylation (OXPHOS) at electron transport chain (ETC) complexes. Detoxify free radicals from ETC complexes reduced by manganese superoxide dismutase (MnSOD) and glutathione peroxidase (GPX) into water (H_2_O). GPX, as a catalase, also regulates the conversion balance of oxidized glutathione (GSH) and glutathione disulfide (GSSG) in mitochondria. Under the circumstance of oxidative stress that is stimulated by insults and exogenous pathogens, various DNA sensors trigger aberrant mitochondrial function by activation of pattern recognition receptors in the cytosol and mitochondria (e.g. retinoic-acid-inducible protein I (RIG-I), melanoma differentiation-associated protein 5 (MDA-5), and Toll-like receptor(TLR)). Expulsion of highly immunogenic Ox-mtDNA out of mitochondria stimulates AMP-activated protein kinase (AMPK), mammalian target of rapamycin complex 1 (mTORC1), Nucleotide-binding oligomerization domain (NOD)-like receptor pyrin domain-containing protein 3 (NLRP3)-mediated IL-1β and IL-18 production, and cyclic GMP-AMP (cGAS) and the cyclic GMP-AMP receptor stimulator of interferon genes (STING)-dependent IFN-α. Under conditions of nutrient deprivation, AMPK inhibits energy-consuming pathways by mTORC1 for mitophagy activation. The HRES-1/Rab4-mediated dynamin-related protein 1 (Drp1) depletion reverses mitochondrial accumulation *via* mTOR-independent pathway. Mitochondrial respiration (ETC), mTORC1, and cGAS-STING signaling pathway serve as potential therapy targets. Several medications that targeting mitochondria metabolism process has been observed the therapeutic clinical effectiveness in SLE, including N-acetylcysteine (NAC), Rapamycin, Metformin, Mito Q, Hydroxychloroquine (HCQ), and Idenenone. The voltage-dependent anion selective channel (VDAC) oligomer inhibitor VBIT 4 can inhibit the release of mtDNA. I, II, III, IV, and V represent the electron transport chain complexes I–V. Glutathione reductase (GR).

The tricarboxylic acid (TCA) cycle occurs in the mitochondria is essential for ATP energy supplement and participates in multiple mitochondria metabolic pathways (13, 14). This review mainly discusses defective mROS clearance for nutrient energy signaling activation (e.g., activation of AMP-activated protein kinase (AMPK) and mammalian target of rapamycin complex 1 (mTORC1)) by regulating the mitochondrial dynamic loop and releasing the antigenicity of the mtDNA to induce the immune responses. Furthermore, preclinical safety therapeutics for the clearance of excessive mROS and mtDNA production were collectively recapitulated in autoimmune diseases, particularly SLE (**Figure 1**).

### Mitochondria dynamics balanced with biogenesis and mitophagy

**Figure 2:**
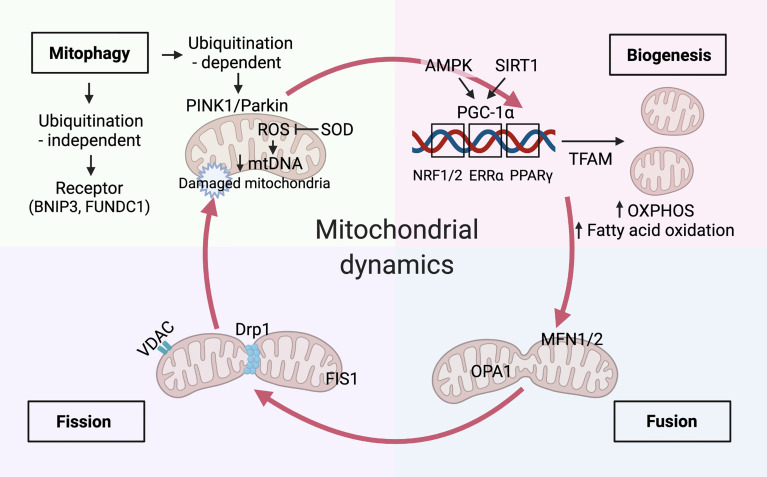
Mitochondria dynamics balanced with biogenesis and mitophagy. Mitochondrial biogenesis. Immune signals of AMP-activated protein kinase (AMPK) and sirtuin 1 (SIRT1) have been reported to increase mitochondrial biogenesis by peroxisome proliferator-activated receptor-γ coactivator-1α (PGC-1α). The master regulator, PGC-1α, drives mitochondrial biogenesis, oxidative phosphorylation(OXPHOS), and fatty acid oxidation by co-activating transcription factors, including Estrogen Related Receptor Alpha (ERRα), peroxisome proliferator-activated receptor (PPAR)-γ, nuclear respiratory factor (NRF)-1 and-2. Mitochondrial transcription factor A(TFAM) aids in the transcription of genes that is essential for balancing mtDNA transcription and clearance. Mitochondrial dynamics. Mitochondrial dynamics of fission and fusion are regulated by dynamin-related protein-1 (Drp1), fission 1 (Fis1) and mitofusin-1 (Mfn1), mitofusin-2 (Mfn2), and optic atrophy 1 (Opa1) respectively. mtDNA extrudes from the mitochondria through pores formed by voltage-dependent anion-selective channel (VDAC) oligomers. Mitophagy activation. The augment of fission promotes mitophagy activation by the PTEN-induced kinase 1 (PINK1)/Parkin-mediated ubiquitination pathway and receptor-mediated pathways, such as BCL2 interacting protein 3 (BNIP3) and FUN14 domain-containing protein 1(FUNDC1). Timely elimination of damaged and aged mitochondria reduces the excessive accumulation of mitochondria reactive oxygen species (mROS), which can be inhibited by mitochondrial enzymes such as superoxide dismutase 2 (SOD2). Damaged mitochondria with decreased mtDNA undergo mitophagy.

Mitochondria continuously undergo a cycle of production (biogenesis), morphology (fusion/fission), and degradation (mitophagy); hence, they are crucial for maintaining cellular homeostasis (15) (**Figure 2**).

The mitochondrial biogenesis and function require peroxisome proliferator-activated receptor (PPAR)-γ coactivator-1 (PGC) family of transcriptional coactivators, including PGC-1α, PGC-1β, and PGC-1–related coactivator(PRC) (16). PGC-1α is a primary mitochondrial biogenesis core regulator that recruits several transcription factors, such as nuclear respiratory factor (NRF)-1, NRF-2, and estrogen-related receptors. It also promotes the assembly fatty acid β-oxidation, OXPHOS and co-activate the mitochondrial biogenesis. Remarkably, NRF-1 and NRF-2 significantly induce the transcription of mitochondrial proteins in the nucleus; among these proteins, the expression of the mitochondrial transcription factor A (TFAM) is essential for balancing mtDNA transcription and clearance (17). PGC-1α plays a complex role in regulating the expression of ROS-scavenging proteins, such as superoxide dismutase (SOD_2_) (18). Furthermore, in response to the cellular energy demands, it activates the key energy sensors AMPK and sirtuin 1 under differential nutritional conditions. Owing to its critical role in cellular energy homeostasis, AMPK participates in various catabolic processes. Indeed, its phosphorylation stimulates mitochondrial biogenesis, autophagy, mitophagy, fatty acid oxidation (FAO), and glycolysis, and induces ATP generation during cellular metabolism (19). We discussed AMPK-mediated regulation of PGC-1α expression in kidney podocytes in the SLE organ damage section (4.1 Mitochondria and Lupus Nephritis). However, AMPK inhibits energy-consuming pathways *via* mTORC1 and ULK1 phosphorylation under nutrient deprivation conditions, consequently activating mitophagy (20). The mammalian target of rapamycin (mTOR) pathway is another nutrient-sensing pathway that regulates mitochondrial biogenesis and activates PGC-1α in response to external stimuli (21). Furthermore, PGC-1α interacts with various transcription factors, such as hypoxia-inducible factor 1 subunit alpha (HIF-1α) that mediates mitochondrial biogenesis and mitophagy (22).

Distinct mitochondrial molecular mechanisms and signaling pathways have been proven to regulate the pathways mentioned above (23). For instance, mitochondrial fusion is primarily mediated by mitofusin 1/2 (Mfn1/2), optic atrophy protein 1 (Opa1), and three GTPases. Mfn1/2 localizes to the outer mitochondrial membrane and facilitates the fusion of adjacent mitochondria by forming homo- or heterodimers. In contrast, Opa1 predominately induces the fusion of the inner mitochondrial membrane in a synchronized manner. Additionally, dynamin-related protein (Drp1) and other receptors play essential roles in regulating mitochondrial fission through phosphorylation and ubiquitination (24). Notably, Drp1 mutation affects mitochondrial fission, ROS generation, and kidney-specific cell damage and effacement, eventually initiating cell apoptosis (25). Mitochondrial fission-mediated fragmentation, concomitant with other mitochondrial damage, initiates mitophagy and mediates mROS production. On the contrary, mitochondrial fusion promotes the exchange and restoration of mitochondrial content by tethering adjacent mitochondria (26). The mitochondrial fission and fusion dynamics modulate a metabolic shift towards cell differentiation. For example, memory T cells have fused mitochondria that favor OXPHOS and FAO, whereas effector T cells enforce fission of punctate mitochondria, promoting the catabolic aerobic glycolysis pathway (27). This suggests that manipulating the mitochondrial structure can determine cell differentiation and, eventually, cell fate.

Mitophagy is closely associated with mitochondrial fission and selectively removes damaged or depolarized mitochondria during lysosomal degradation (28). Similar to autophagy, mitophagy is a catabolic process that is initiated by the recruitment of autophagy machinery proteins to activate the mitochondrial surface. Thereafter, autophagosomes enclose mitochondria and transport them to lysosomes for degradation (29). Two major degradation processes enable the removal of defective mitochondria to maintain the homeostasis of cell function and metabolism. For instance, ATPases specifically degrade senescent and damaged mitochondria into small peptides; however, these mitochondria can also be eliminated in lysosomes through mitophagic degradation (30). Under cellular stress stimuli, mitophagy selectivity initiates multiple cell death signals through the PTEN-induced kinase 1 (PINK1)/Parkin-mediated ubiquitination pathway and receptor-mediated pathways, such as BCL2 interacting protein 3 and FUN14 domain-containing protein 1 (31, 32). PINK1 and Parkin-mediate ubiquitination signaling play crucial roles in the interplay between mitophagy and mitochondrial dynamics. Hence, timely elimination of damaged and aged mitochondria reduces the excessive accumulation of mROS, avoiding triggering the cytoplasmic DNA sensor by releasing the mtDNA (33). Mitochondria are endosymbionts evolved from bacteria. The mtDNA and N-formyl peptides are two mitochondrial components that represent mitochondrial damage-associated molecular patterns (DAMPs), serve as the innate immune response to produce inflammatory cytokines and chemokines *via* Toll-like receptors (TLR), absent in melanoma 2 (AIM2), nucleotide-binding oligomerization domain (NOD)-like receptors (NLRs), and retinoic acid-inducible protein I (RIG-I)-like receptors (RLRs) (33). Beyond the clearance capability of mitophagy, damaged mitochondria trigger the innate immune response by releasing mitochondrial DAMPs to recognize pattern recognition receptors and G-protein-coupled formyl peptide receptors, which explains the stimulation of a pro-inflammatory response in the innate immune system under microbiology-free condition (34).

## Mitochondrial dysfunction in SLE

### The vicious cycle of redox disturbance

Under physiological conditions, mitochondria generate low levels of mROS to modulate various signaling pathways and cellular metabolisms. However, mROS levels beyond a threshold can be pathogenic, causing mitochondrial dysfunction and further cellular damage (35). Glutathione peroxidase (GPX), as one of the antioxidant enzymes to balance between glutathione and glutathione disulfide, detoxifies lipid peroxides and scavenges ROS from the mitochondria (36). Under the oxidative stress condition, mtDNA can be extruded from the mitochondria into the cytoplasm (4). A recent study has discovered that the formation of 8-hydroxy-2-deoxyguanosine (8-OHdG), which is recognized as an indicator of oxidant-induced DNA damage, is greatly eliminated upon the decline of the mtDNA copy number in SLE patients (37). On the other side, highly immunogenic Ox-mtDNA induces mROS overproduction and stimulates an inflammatory response (38). In addition, defective mitochondrial antioxidant enzymes in the immune cells of SLE patients can further aggravate oxidative stress in a vicious cycle (38). Therefore, oxidative stress-mediated mitophagy has been recognized to play a critical role in genetic predisposition and immune cell activation in SLE.

### Genetic mechanism of mitochondrial dysfunction

Genetic predisposition along with many other etiological factors contributes to the pathogenesis of SLE. Multiple genome-wide association studies have identified several mitochondrial single nucleotide polymorphisms as responsible for conferring susceptibility towards SLE (39). For example, ATP synthase 5/6, displacement-loop, D310, NADH dehydrogenase subunit (ND) 1, and ND2 have been demonstrated to be involved in mitochondrial biogenesis and mitophagy, proving that mtDNA polymorphisms confer susceptibility to SLE (39–42). As known, pathogenic autoantibodies and the deposition of immune complexes in tissues cause multiple organs damage in SLE. Autoantibodies against various mitochondrial components indicate that mitochondria are key antigenic stimulants that incite an immune response in SLE. Various autoantibodies, such as anti-mitochondrial, anti-whole mitochondrial (43), anti-mtDNA (44), mitochondrial-RNA (45), and anticardiolipin, have been generated by targeting distinct mitochondrial components, including the mitochondrial surface, mtDNA, mitochondrial RNA, and mitochondrial inner membrane, respectively. These findings suggest that mitochondria are an important source of autoantigens that contribute to SLE pathogenesis. Furthermore, accumulating evidence suggests that mitochondrial dysfunction and abnormal mitophagy result in redox disturbance of mtDNA, overproduction of mROS, an imbalance of oxidative stress, and activation of inflammatory pathways. All these factors are involved in the initiation and development of SLE (46).

### Mitochondrial dysfunction in immune cells

Mitochondria are hypothesized to originate from endosymbiotic bacteria. Additionally, self-energy modulation in the mitochondria is essential for mitochondrial metabolism and host immune response signaling cascade (47). Mitochondrial dysfunction-induced redox disturbance differentially activates various inflammatory pathways in adaptive and innate immune cells. Studies have investigated multiple cellular signaling pathways that regulate mitophagy in SLE, including the mTORC1, AMPK, NLR family pyrin domain-containing 3 (NLRP3) inflammasome, and cGAS–STING pathways (48–51) (**Figure 1**).

#### Mitochondrial dysfunction in adaptive immune cells

Mitochondrial dysfunction has been detected in lupus T cells and is characterized by excessive mROS production, elevated transmembrane potential, downregulated mitophagy, and reduced glutathione levels (52–54). Moreover, oxidative stress impairs proximal T cell receptor (TCR) signaling, pathway activation, and exhaustion-associated gene expression program activation (55). Furthermore, chronic antigen stimulation induces irreversible T cell exhaustion and impairs mitophagy, causing mROS overproduction and autoantigen release (56).

Multiple signaling pathways enable the sequestration and successful clearance of damaged mitochondria by mitophagy, suppressing mtROS accumulation (57). Redox-dependent activation of the mTOR pathway plays an essential role in altering TCR signal transduction and T cell differentiation in SLE patients (48, 58). Additionally, it is believed that mitochondrial localization to an immune synapse is required for T cell activation. However, mTOR activation decreases T cell surface receptor/CD3ζ chain levels and in compensation augments tyrosine-protein kinase and Fcgamma chain receptor (FcγR) levels. Consequently, this increases the calcium flux in lupus T cells through HRES-1/Rab4-dependent lysosomal degradation (48). The aberrant Ca^2+^ alteration may account for the inappropriate activation in lupus T cells. Notably, upregulated expression of the small GTPase HRES-1/Rab4 depletes the Drp1 levels, leading to decreased mitophagy in CD4+ T cells in lupus. In contrast, Rab4 inhibited with 3-pyridinyl ethylidene hydroxyl phosphonocarboxylate (3-PEHPC) restores Drp1 function *via* the mTOR-independent pathway, reversing mitochondrial accumulation in lupus T cells (59).

Increased calcium flux also promotes oxidative stress-dependent T cell activation. For instance, the calcium-mediated phosphatase calcineurin dephosphorylates nuclear factor of activated T cell (NFAT). Intranuclear NFAT, accompanied by other transcription factors such as AP-1, NF-κB, and Oct-1, promotes the expression of the inflammatory cytokine interleukin (IL)-2 (54). Of note, Rab4A inhibition improves impaired mitophagy of lupus T cells, activation of T cells, and production of autoantibodies in SLE. This demonstrates that recovery of mitochondrial dysfunction caused by insufficient mitophagy and mROS overproduction normalizes the phenotypes of T cells in SLE. Furthermore, few clinical trials have demonstrated that either inhibiting mTORC1 by rapamycin (sirolimus) (60) or supplementing N-acetylcysteine (NAC), a glutathione precursor and an antioxidant (58), is therapeutically effective and improved systemic inflammation in patients with SLE. Interestingly, mitochondrial accumulation depletes memory T cells and regulatory T (Tregs) cells in SLE (60). Indeed, decreased number of Treg cells fail to maintain self-tolerance and immunosuppressive function in SLE, whereas supplementation of Treg cells normalizes the inflammatory response and SLE pathology in lupus mice (61). With regard to mitochondrial energy metabolism, these cells have a higher requirement for OXPHOS *via* AMPK activation than the T helper 17 (Th17) cell subset (62). Metformin, by targeting AMPK activation, can optimize the immunoregulatory effect of Treg cells by enhancing STAT1 expression in an AMPK-dependent manner in SLE (63).

Little is known about the mitochondrial immune metabolism of B cells in SLE. Nonetheless, induced differentiation of CD27^+^IgD^+^ unswitched memory B cells into D27^hi^CD38^hi^ plasmablasts is accompanied by activation of mTORC1 (64). In fact, increased activation of mTORC1 has a positive correlation with the overproduction of autoantibodies and cytokines in the B cells in SLE (64, 65). Remarkably, suppression of hyper-responsiveness in B cells was observed upon rapamycin treatment in SLE (66). with consistency, suppressing mTOR activity by deletion of RAPTOR, an essential signal adaptor for mTORC1, blocks plasma cell differentiation in a mouse model of lupus (67). Previous evidence has also revealed that long-lived plasma cells require quiescent and slow ATP generation through AMPK signaling and mitochondrial OXPHOS (68). Metformin has been demonstrated to ameliorate SLE manifestations by preventing B cell differentiation into plasma cells and germinal center expansion by altering AMPK–mTOR–STAT3 signaling (69).

#### Mitochondrial dysfunction in innate immune cells

Neutrophils and plasmacytoid dendritic cells (pDCs) produce IFN-α under the stimulation of nucleic acid–protein immune complexes (70). Of note, excessive IFN-α production occurring in more than half of all SLE patients stimulates the release of interferongenic DNA (including mtDNA) from neutrophils, forming an autoantigen/antibody immune complex (71). However, exposure to the IFNα/RNP immune complex disrupts mitochondrial degradation, leading to the accumulation of abundant Ox-mtDNA within mitochondria and eventually out of mitochondria, which would be a potent pDC stimulator (49). Owing to the presence of the Ox-mtDNA autoantibody, mtDNA is potent antigen that stimulates autoantibody production in SLE (45). Furthermore, the common oxidative DNA damage marker 8-OHdG is elevated in the blood cells of SLE patients (37). mtDNA served as a DAMP induces the production of IFN-α, which activates the cytosolic DNA-sensing cGAS-STING (72), and NLRP3 signaling pathway (51). Defective mitophagy and consequent mtDNA-dependent DNA sensor activation might conceivably be a fundamental event in SLE pathogenesis (3).

The formation of neutrophil extracellular traps (NETs), which are extrusions of genomic DNA and chromatin to kill invading pathogens (73). The ribonucleoprotein immune complex and IFN-α induce spontaneous NETosis through cGAS–STING pathway activation, mitochondrial hyperpolarization, and mROS overproduction in SLE (74). Notably, mROS-mediated Ox-mtDNA enclosed within NETs that are expelled to the neutrophil cell surface triggers IFN production. Thus, mROS inhibition reduces NETosis by inhibiting mitochondrial respiration in lupus mice. This reinforces the notion that mitochondrial functioning and ROS productivity drive SLE pathogenesis (75).

In addition, high mROS levels and increased oxidative stress have been detected in SLE monocytes exhibiting IFN-α signature. Defection of IFN-α-mediated mtDNA degradation leads to a high mitochondrial membrane potential in SLE monocytes, promoting their autoreactivity in a STING-dependent manner (76). Moreover, the differentiation of circulating monocytes into autoinflammatory dendritic cells in the presence of IFN-α promotes the activation and expansion of autoreactive lymphocytes, partially mediating an adaptive immune response (77). Notably, pDCs are the major producers of IFN-α in SLE, and IFN-α production from pDC mediated by TLR9 is reduced by mTOR inhibitor, which regulates pDC differentiation and activation by modulating mitochondrial biogenesis and energy metabolism (78). Therefore, normalization of mtDNA and repairment of mitophagy by an antioxidant treatment are promising therapeutic strategies for SLE treatment (76).

NLRP3 and AIM2 are DNA sensors that along with apoptosis-associated speck-like protein and pro-caspase-1 (component of the NLRP3 inflammasome) trigger the production of several downstream pro-inflammatory cytokines (e.g., IL-1β and IL-18) and promote pyroptosis (79). Although multiple models of NLRP3 inflammasome activation have been identified in autoimmune diseases, the most common model comprises NLRP3 inflammasome activation with ROS production (80, 81). Clearance of damaged mitochondria by decreasing oxidative stress signals over time and increasing mitophagy physiology are necessary for limited NLRP3 activation. In addition, mROS and mtDNA are crucial for optimal NLRP3 inflammasome activation and concomitant calcium influx (82). Mitochondria are essential for intracellular calcium storage and are key modulators of mitochondrial homeostasis. Of note, calcium influx promotes the release of mROS and mtDNA to amplify NLRP3 activation in a feedback loop (83). NOD2/RIPK2-dependent mitophagy and LC3B/Beclin 1-mediated autophagy contribute to the clearance of damaged mitochondria and thus negatively regulate NLRP3 inflammasome activation (82, 84).

Several studies have also indicated that NETs-mediate the activation of the NLRP3 inflammasome in macrophages of lupus patients, subsequently promoting IL-1β production *via* ROS and K^+^ efflux (85). Importantly, insufficient clearance of damaged mitochondria causes excessive mtDNA, mROS, and cardiolipin externalization and that of other stimulators (e.g., K^+^ efflux), thereby activating the NLRP3 inflammasome (86). However, administration of mROS scavengers significantly decreases inflammasome-related gene expression and mature IL-18 expression in lupus mice (75). Conversely, studies reported that loss-of-function mutations of inflammasomes are correlated with disease development in SLE patients (87). The possible explanation is that homeostatic inflammasome function is required for the equilibrium of protective and destructive immunity. In addition, defective lysosomal degradation of autophagosomes and their cargo in macrophages fail to clear mtDNA and lysosomal TLR7 activation. Notably, lack of mitochondria-related GTPases, e.g. the IRGM1, could lead to sustained activation of IFN, consequently induce cGAS–STING axis activation and autophagy inhibition in various autoimmune diseases (88).

## Mitochondrial dysfunction damages organs

**Figure 3:**
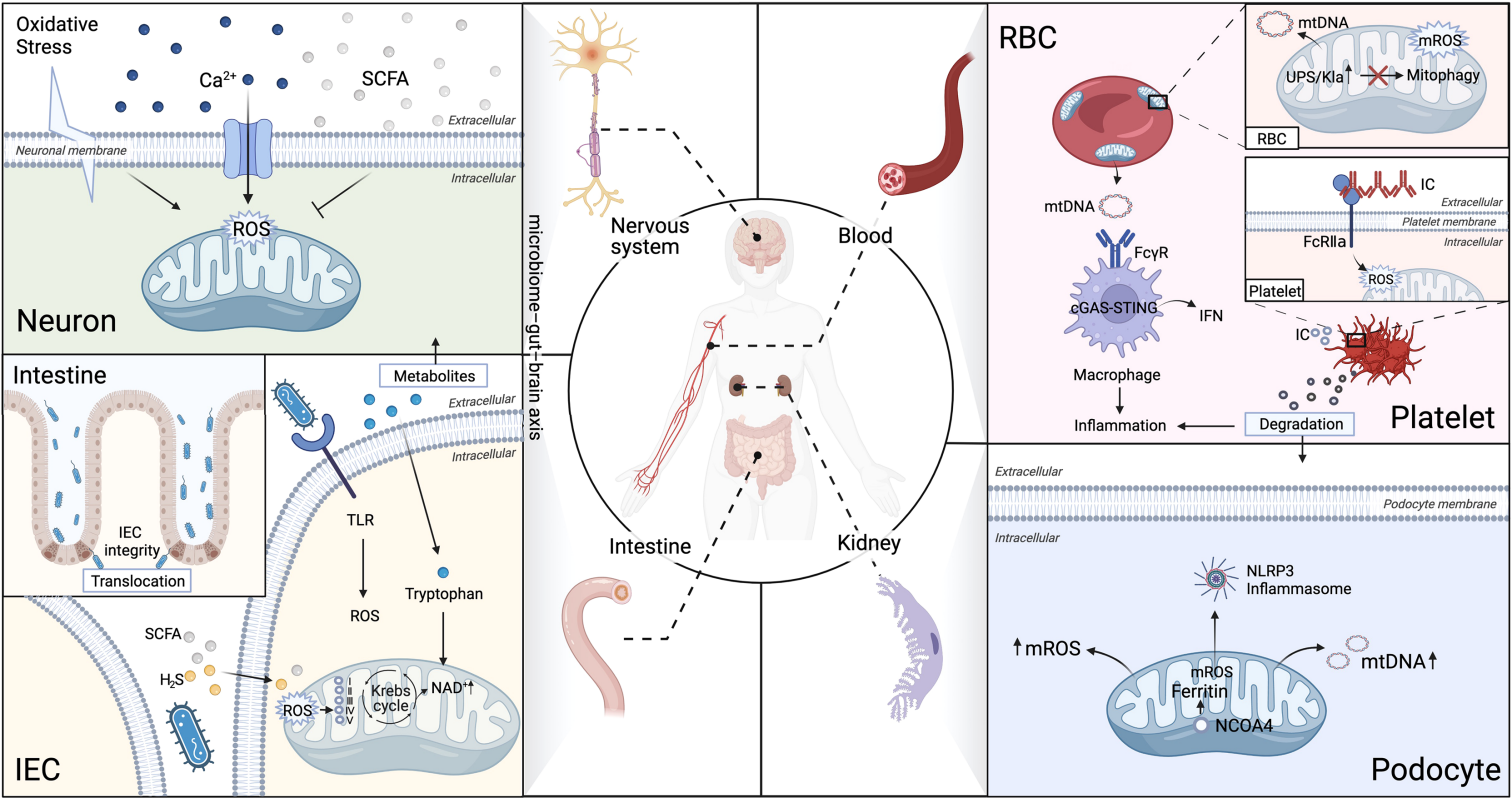
Aberrant mitophagy and redox disturbance cause multiple organs damage in SLE. SLE is a heterogeneous systemic autoimmune disease, affected multiple organs, such as kidney, nerves, gut and hematologic (non-immune cells). **(A)** Brain-Gut axis is novel therapeutic strategy on modulation of mitochondrial dyshomeostasis by neutralizing excessive ROS in microglial cells in neuropsychiatric SLE (NPSLE). **(B)** Extracellular mitochondria (specifically platelets mitochondria and erythrocytes) serve as an immunogenic DNA, activated the cGAS–STING pathway, consequently trigger IFN-α production through binding to FcγRIIA in SLE. **(C)** The microbiota metabolites can directly interfere with the mitochondrial redox status in the host cells. Microbiota metabolites, such as hydrogen sulfide (H_2_S), short-chain fatty acids (SCFAs), and tryptophan, play an essential role in regulation of intestinal epithelial cells **(IEC)** integrity in SLE. **(D)** Impaired mitochondrial degradation in glomerular and tubular cells causes proteinuria and renal failure in LN. Iron loss-induced mitophagy is associated with the mitochondrial ferritin by nuclear receptor coactivator 4(NCOA4). Iron-induced injury of glomerular and tubular cells has been attributed to excessive mROS production, which contributes to drive NLRP3 inflammasome activation in SLE podocytes. Ubiquitin-proteasome system (UPS), Five respiratory complexes (complexes I–V), tricarboxylic acid (TCA) cycle.

Target organs of SLE, especially kidney, nerves, and gut, require high level of energy supply to drive their function and are more susceptible to oxidative stress and subject to chronic inflammatory injuries (1). Moreover, it is being increasingly evident that impaired mitochondria are central mediators of injury in different tissues and organs in SLE (46), which will be discussed in-depth in the subsequent section and summarized in **Figure 3**.

### Mitochondria and lupus nephritis

The kidney is one of the most energy-consuming organs in human body and the mostly attacked organ in lupus (89). Indeed, glomerular podocytes and tubular epithelial cells require a bundle of mitochondria to provide sufficient energy to remove waste from the blood and maintain the electrolyte balance (90). Current evidence suggests that impaired mitochondrial degradation in glomerular and tubular cells are involved in LN pathogenesis and renal damage (91). Targeted therapies against impaired mitophagy have been designed to prevent LN (91–93).

Dysfunction of podocytes with abnormal permeability and viability and aggravated podocytes apoptosis by pathogenic IgG and IFN-α have been found in LN patients and lupus mice, which could be partially restored by activation of autophagy *via* mTORC1 inhibition (91). Recent evidence demonstrates that lupus mice with severe proteinuria had low autophagosomes in the podocytes with significant foot process effacement and fusion, while lupus mice with mild proteinuria had a high number of autophagosomes observed with slight podocyte foot fusion. Podocyte function is regulated by an imbalance in mitochondrial division and fusion and activates mitophagy to remove damaged mitochondria *via* Mfn1 and Drp1 expression. However, once decompensated, mitochondrial dysfunction leads to a vicious cycle of defective mitophagy in LN (94). Therefore, further investigation of systemic and local mitophagy mechanisms is required to elucidate LN.

Since glomerular and tubular cells are metabolically active, they are particularly susceptible to oxidative stress-mediated mitophagy. The uptake of filtered iron and the high mitochondrial content are essential for iron metabolism, which is an under-investigated driver of LN (95). Podocytes serve as the primary components of iron uptake in glomerular epithelial cells. Indeed, they take up hemoglobin and transferrin *via* megalin–cubilin complex-mediated endocytosis and store the absorbed iron as ferritin (96). Notably, recent evidence has identified novel mechanisms of mitophagy independent of the PINK1/Parkin pathway (97). For instance, mitophagy induced by iron chelator-mediated iron loss is associated with increased expression of mitochondrial ferritin. In fact, the mitochondrial ferritin interacts with nuclear receptor coactivator 4, an autophagic cargo receptor, triggers mitophagy and regulates the expression of antioxidants and detoxifying enzymes *via* HIF-1α specific protein 1 axis (92). Furthermore, studies have demonstrated that excessive dietary iron intake may aggravates human lupus, whereas iron infusion increases disease progression and worsens symptoms (98). Renal iron accumulation have been reported in different strains of lupus mice, and hepcidin could ameliorates LN development in MRL/lpr mice by regulating iron metabolism (99). Treatment with deferiprone, a U. S. Food and Drug Administration-approved iron chelator, significantly ameliorates pathogenic anti-dsDNA IgG expression and renal injury. Although deferiprone has been authorized for only limited diseases, its ability to interfere with iron loss‐induced mitophagy highlights its therapeutic potential in treating SLE and thus deserves further investigation (95). In lupus-prone mice with neutrophil-specific glutathione peroxidase-4 (GPX4) haploinsufficient, treatment with a specific ferroptosis inhibitor *via* GPX4, the component most downstream to the ferroptosis pathway, significantly ameliorated disease severity. This further proves the feasibility of manipulating iron metabolism in SLE treatment (100).

Remarkably, the molecular mechanism of iron-induced injury in glomerular and tubular cells has been attributed to mROS production (93). Indeed, excess mROS activates the NLRP3 inflammasome by upregulating IL-1β and IL-18 levels, further exacerbating inflammatory reactions within these cells (51, 93). Studies have also determined the activation of the podocyte NLRP3 inflammasome in both lupus-prone mice and LN patients (101, 102). Mitochondrial ROS inhibitors can suppress the activation of the NLRP3 inflammasomes alleviate the podocyte lesions and proteinuria (101, 103).

To date, there has been a lack of mitochondrial antioxidant therapeutics developed in preclinical studies. Notably, supplementation of coenzyme Q_10_ (CoQ_10_), a potent antioxidant, significantly reduces tacrolimus -induced oxidative stress and mitochondrial membrane potential in proximal tubular cells. Thus, this can be a promising approach to reduce nephrotoxicity *via* regulation of mitochondrial function (104). More potential targets treatment beneficial for mitochondrial function and structure, and preventing oxidative injuries are expected.

### Mitochondria and microbiome dysbiosis

It has been widely proposed that mitochondria and the gut microbiome have a shared phylogenetic history, and their crosstalk regulates cellular homeostasis and metabolism (47). Furthermore, increasing evidence suggests that microbiota and their metabolites directly interfere with the mitochondrial respiratory chain and maintain the redox status in host cells (105).

Emerging evidence suggests that a disturbed gut microbiota is an essential pathogenic mechanism in SLE development (106, 107). Owing to their tight junctions and coated mucus layers, host intestinal epithelial cells (IEC) act as frontline machinery that partition the host inner organs from the gut microbiota in the lumen (108). Gut microbiome regulates mitochondrial energy production *via* biosynthesis and mitophagy, whereas mitochondrial homeostasis in IECs maintains intestinal barrier integrity against pathogens (109, 110). Of note, excessive mROS levels induce a redox imbalance and modify phosphorylation of tight junction proteins (e.g. occludin and ZO-1), and disrupt the tight junctions of the intestinal epithelial barrier (111). However, studies have noted that treatment with the mROS scavenger mitoquinone (MitoQ) and the antioxidant NAC restores mitochondrial ATP synthesis and enhances epithelial cell mitophagy *in vivo* and *in vitro* (112, 113).

The interaction between epithelial cell mitophagy and disturbed microbiota in the gut of SLE patients has attracted increasing attention (114). For example, studies have discovered that microbial metabolites, such as hydrogen sulfide (H_2_S), short-chain fatty acids (SCFAs), and tryptophan, in particular, considerably regulate mitophagy in SLE (114–116). Indeed, a previous study has demonstrated that several intestinal pathogens (e.g., *Escherichia coli* and *Salmonella* spp.) exhibiting degradation of sulfur amino acids produce a large amount of H_2_S (117). Recently, a study has detected detrimental responses to excessive intestinal H_2_S, which inhibits complex IV of the mitochondrial ETC during inflammation, reduced SCFA production and damage gut epithelial barrier integrity (115, 118). Increased intestinal permeability and gut microbes translocation have been eventually proposed contributing to the pathogenesis (119). Indeed, translocation of *Enterococcus gallinarum* and *Lactobacillus reuteri* into various organs has been independently implicated in different mouse models of lupus (120, 121).

Microbial fermentation of indigestible dietary fiber produces SCFAs that are primarily composed of acetate, propionate, and butyrate. These compounds are capable of regulating mitochondrial activity (118). Generally, Firmicutes are the principle producers of butyrate, whereas *Bacteroidetes* primarily produce acetate and propionates (122). Accumulating evidence has revealed that gut dysbiosis causes a reduced ratio of Firmicutes/Bacteroidetes (F/B) in SLE patients (123). Nevertheless, butyrate treatment has been demonstrated to significantly alter the microbiome diversity and normalizes the F/B ratio in lupus-prone mice (114). A recent study has reported that activated mitochondrial Drp1 reduces the production of SCFAs and perturbs the relevant microbes in a ROS-specific manner, thereby substantially disturbing intestinal homeostasis (124). Intriguingly, butyrate serves as an important fuel for intestinal cells and participates in the TCA cycle by modulating the mitochondrial ETC and mROS clearance (125). Moreover, sodium butyrate alleviates H_2_O_2_-induced oxidative stress and intestinal epithelium injury *via* the AMPK-mitophagy-dependent pathway. However, treatment with an AMPK inhibitor weakens this positive effect of sodium butyrate on mitophagy, highlighting the importance of sodium butyrate in maintaining mitophagy homeostasis (126). Frontier research on the mitochondria and gut microbiome nexus further illustrates that the AMPK activator metformin can enhance SCFA production (125). These previous data suggest that SCFA modulates gut microbiome diversity and is beneficial for mitochondrial homeostasis in intestinal epithelial cells, making it a complex yet fascinating target for lupus studies.

An abundance of the microbial metabolite tryptophan has been observed in the gut of SLE patients and lupus-prone mice and is considered to shift the composition of the gut microbiome (123, 127, 128). Furthermore, fecal transfer from lupus mice fed with a high-tryptophan diet compared to that from mice fed with a low-tryptophan diet is more likely to induce autoimmunity in Germ-free mice. Consequently, this strengthens the link between gut microbiota and tryptophan metabolism in the lupus phenotype (128). Remarkably, tryptophan is metabolically processed through the kynurenine pathway to produce nicotinamide adenine dinucleotide, which is essential for the mitochondrial redox balance (116). However, the complex link microbial metabolites and mitochondrial functions is just at its beginning to be recognized (118). Owing to the evidence provided by current studies on the direct association between mitochondria and gut microbiota in SLE pathogenesis, increasing focus is being directed toward normalizing defective mitophagy by influencing the gut microbiome diversity and microbial metabolites. Notably, probiotics, diet, and/or fecal transplantation are emerging strategies for maintaining redox balance and mitophagy in SLE (129). Nonetheless, multiple interactions of gut microbiota metabolites and mitochondria in SLE require further investigation.

### Mitochondria and lupus hematological damage

Mitophagy mediates the elimination of organelles that are essential for regulating erythroid maturation. Previously, a study has demonstrated that a deficiency of Nix, a Bcl-2 family member, reduces mature erythrocyte levels and downregulates compensatory expansion of erythroid precursors by regulating the mitochondrial membrane potential (90). A recent study has indicated that over one-third of mature erythrocytes in SLE patients contain mitochondria (130). In this regard, certain SLE patients exhibit a dysfunctional ubiquitin-proteasome system (UPS) that fails to eliminate mitochondria in the mature erythroid cells. It has been proposed that defects in the HIF-1α-mediated metabolic regulation pathway prevent UPS activation and mitophagy initiation, leading to the accumulation of erythrocytes containing mitochondria (131). These mitochondria-containing erythrocytes produce interferongenic mtDNA that serve as immunogenic DNA in SLE, inducing IFN-α production through the activation of the cGAS–STING pathway in macrophages (132).

Presence of antiphospholipid antibodies is prothrombotic and associates with risk of thrombosis. Emerging studies have reported that increased anti-dsDNA autoantibodies or dsDNA immune complexes strongly promote platelet activation and thrombosis events by *via* binding to the FcγRIIA (133). Despite the sources of circulating autoantigens in SLE have not been completely elucidated. New evidence has indicated that mtDNA and mitochondrial extrusion *via* platelet activation through FcγRIIA stimulation induces a circulatory autoantigenic load that ultimately leads to SLE pathogenesis (134, 135). Given their abundance in the blood, platelets are a primary source of mitochondria. In addition, platelet-derived IgG-containing microparticles, mitochondria, mtDNA, and cytokines are targets of autoantibodies to form immune complexes in SLE. The DNA-containing immune complexes captured by Fcγ receptor stimulate multiple intracellular signaling pathways for IFN-α production in SLE, including the TLR9, RLRs, and cGAS–STING pathways (70, 134, 136). Since platelets have crucial roles in an immune response, preventing platelet activation by releasing microparticles and mitochondrial antigens has emerged as a therapeutic strategy for SLE treatment (137).

### Mitochondria and neuropsychiatric SLE

Neuropsychiatric (NP) involvement and cognitive symptoms are some of the most severe manifestations of SLE. These symptoms are induced by multiple immune complexes deposition and by pro-inflammatory cytokines/chemokines (138). However, the mechanism underlying brain abnormalities in NPSLE remains largely unknown, and effective strategies to ameliorate neuropsychiatric disorders are limited.

Since neurons require high energy supplied by healthy mitochondria to get excited, the brain is very sensitive to dyshomeostasis of mitochondrial redox-sensitive signaling (57). Positron emission tomography implemented by a pilot study has revealed that altering the distribution of a mitochondrial translocator protein in the brain significantly affects the cognitive functions of SLE patients (139). The microglia cells are stimulated by pro-inflammatory cytokines to cause impairment in axonal transmission and mitochondrial dysfunction (140, 141). The swollen and vacuolated mitochondria cause Ca^2+^ dysregulation and caspases signal activation (140, 142). However, a therapeutic strategy of neutralizing excessive ROS in microglial cells to modulate mitochondrial dyshomeostasis requires further investigation in NPSLE patients.

Inspired by the gut-brain axis concept, potential neuromodulatory mechanisms of gut microbial metabolites have been extensively studied. Evidence indicates that metabolites influence the neuro microenvironment by modulating mitophagy and microglial activation (143). Notably, SLE patients exhibit lower levels of butyrate, the mostly studied microbial metabolites. Studies have demonstrated that butyrate induces transforming growth factor (TGF)-β production and is essential for microglial maturation and function *in vivo* (144, 145). Additionally, butyrate suppresses the side effects of LPS and restraining the mitochondrial function of oligodendrocytes in a mitochondrial redox-dependent manner. Thus, it is considered to be a key mediator of NP-SLE (146, 147). Moreover, gut dysbiosis and neurotoxic substance production (e.g., amyloid proteins and lipopolysaccharides) triggers microglial activation and inflammation. Several studies have also reported that increased kynurenine serum levels are positively correlated with neurological manifestations of SLE (128, 148, 149). Indeed, a kynurenine-derived metabolite, quinolinic acid, influences neuronal activities and causes neurotoxicity (150, 151). More intensive investigation into the mechanism underlying the mitochondrial redox balance in the pathogenesis of NPSLE is expected.

## Therapeutics targeting the mitochondria

Given the plenty of studies elucidating the essential role of mitophagy, mitochondria have emerged as important pharmacological targets. Thus, novel intervention strategies, such as treatment using antioxidants, metabolic rescue of autolysosomal degradation, and repurposing of conventional drugs, are possible options to counteract or fine-tune the current treatment for SLE (152).

### Sirolimus

Sirolimus is an mTOR inhibitor that naturally occurs as an antifungal agent and was first discovered in *Streptomyces hygroscopicus*. It exhibits anti-tumor, anti-proliferative, anti-fibrotic, and immunosuppressive effects (153–155). Sirolimus blocks both mTORC1 activation and T-cell hyperactivity, and its effectiveness has been demonstrated in lupus mice by normalizing proteinuria, protecting renal function, reducing anti-dsDNA titers and inhibiting antiphospholipid antibody production (156, 157). In 2018, a single-arm, open-label trial further verified the role of mTOR inhibition by sirolimus in treating SLE to recover mitochondrial dysfunction (60). Both the off-label drug use and subsequent open-label phase 1/2 clinical trial revealed that sirolimus effectively reduced SLE disease activity (60, 158), and could be an effective therapeutic medication for SLE. Nonetheless, further randomized controlled trials of sirolimus in SLE are warranted.

### NAC

The glutathione precursor NAC is an antioxidant and ROS scavenger that inhibits mTORC1 and suppresses T cell overactivation (53). By disrupting the mTOR pathway and decreasing ROS levels, NAC displayed beneficial efficacy in disease activity of lupus patients (58). As was aforementioned, impaired mitochondrial function, accompanied by increased mitochondrial transmembrane potential and sustained hyperpolarization, potentiates the death of pro-inflammatory T cells in SLE (53). Importantly, a randomized, double-blind clinical trial has proven NAC to be an effective inhibitor of mTOR activity and a regulator of the mitochondrial transmembrane potential in SLE (58). As it is an ROS scavenger and a glutathione precursor, NAC decreases ROS levels while increasing glutathione levels in peripheral lymphocytes (159). Moreover, NAC inhibits mitochondrial ETC complex I, reduce the oxidative stress (159, 160), diminish serum anti-dsDNA antibodies and improves lupus nephritis as well as prevent disease flares in lupus mice (161).

### CoQ10

CoQ10 is a potent antioxidant that prevents the oxidation of cell membranes and cellular components by scavenging free radicals in the cytoplasm. Furthermore, it plays a key role in electron transport from complexes I and II to complex III in the mitochondria (162). It is well recognized that mROS production triggers immune responses against SLE through NET formation (74). A synthetic analog of CoQ10, MitoQ, effectively suppresses autoimmune inflammation in SLE by inhibiting mROS generation. This further downregulates granulocyte NETosis, reduces disease activity, inhibits IFN-α responses, and suppresses immune complex formation in the kidneys in a lupus mouse model (74, 163). Furthermore, another synthetic quinone analog of CoQ10, idebenone, has shown effectiveness in improving immune dysregulation, preventing organ impairment, and restoring mitochondrial abnormalities (75).

### Metformin

Metformin is a first-line antihyperglycemic medication for type II diabetes mellitus; however, it is also a promising therapeutic agent for SLE with immunomodulatory properties. Studies have observed the normalization of T-cell mitochondrial metabolism (including mitochondrial oxidative stress and redox balance) in SLE patients with metformin treatment (164–166). Remarkably, add-on metformin treatment for mild or moderate SLE decreases clinical flares, and reduces glucocorticoid dose (167). Furthermore, metformin selectively inhibits mitochondrial complex I and reduces NADPH oxidase activity. While it significantly downregulates DNA release from NETs by suppressing ROS production (168), it also activates AMPK and suppresses OXPHOS in different immune cells (169). Additionally, it has been proven to be effective in inhibiting B cell differentiation into plasma cells in the germinal center of lupus-prone mice, reduction of NLRP3 inflammasome activation mediated by AMPK, and blockade of pyroptosis of tubular epithelial cells in pristane-induced lupus mice (69, 170). Importantly, the beneficial effects of metformin have been confirmed *via* a *post-hoc* pooled analysis, demonstrating its potential in improving SLE disease activity and decreasing flare risks, particularly in serologically quiescent SLE patients (171).

### Chloroquine

Hydroxychloroquine (HCQ) and chloroquine (CQ) have clinical applications for prevention and treatment of malaria and have been repurposed to treat milder manifestations of SLE, such as skin rash and arthritis (172). Although their precise mechanisms of action remain to be completely elucidated, it has been reported that HCQ inhibits the mitochondrial antioxidant system induced by TCR crosslinking, consequently increasing mROS levels and reducing CD4+ T cell proliferation through oxidative stress (173). Furthermore, HCQ exhibits immunomodulatory properties that downregulate the constitutive activation of TLR7 and TLR9 in antigen-presenting cells, and inhibited the cytokine production thereby (174). Another potential mechanism of HCQ and CQ is to regulate cGAS activity by interfering with its binding to cytosolic DNA, eventually reducing pro-inflammatory cytokine production (175).

### Potential mitochondrial therapeutic targets in SLE

A few potential mitochondria-targeting chemicals have emerged as beneficial therapeutics in lupus mice based on the normalization of mitophagy metabolic pathways. For instance, studies have shown that treatment with 3-PEHPC, an inhibitor of Rab geranylgeranyl transferase, restores Drp1 expression, enables mitochondrial accumulation, and prevents Antinuclear antibody production and nephritis in MRL/lpr mice. Additionally, these 3-PEHPC-treated mice exhibit altered inflammatory cytokine profiles and low IL-10 expression post-treatment (59). Another potential mitochondria-targeting agent is pioglitazone, a PPARγ agonist, induces the disassembly of complexes I and III in the mitochondria, depleting cellular ATP production by upregulating the mitochondrial respiratory chain subunit gene (176). Interestingly, murine lupus models treated with pioglitazone or rosiglitazone have displayed positive responses regarding their inflammatory pathways and disease activity (177, 178). Furthermore, KN-93 is another potential therapeutic agent that inhibits CaMK4 *via* the AKT/mTOR signaling pathway (179). Notably, it can increase Treg cells and decrease the differentiation of Th17 cells in lupus-prone mice, which contribute to suppresses the development of glomerulonephritis and skin diseases (180, 181). cGAS–STING pathway is another possible strategy to develop for SLE patients. Moreover, a potent small-molecule inhibitor of STING, identified as H-151, has been proven to inhibit both murine and human STING activation. Therefore, it is under significant clinical consideration (182).

Only limited mitochondria-targeted drugs have been translated in the clinical application of SLE. With the further understanding of the unique energy metabolism feature of mitochondria in a distinct population of immunocytes, tissue or organ-specific therapeutics of mitochondria-targeting intervention may provide promising strategies for SLE treatment. Due to the critical role of mROS in many pathophysiological processes, the majority of mitochondria-targeted drug focus on antioxidants (183). The antioxidant lipophilic cations have great potential for the treatment of a wide range of pathologies. However, there are still some other prospects for new mitochondrial modulation drugs, depending on increasing lipophilic cation moieties, targeting cardiolipin, and aiming on mitochondrial targeting signal peptides (184). Besides to mitochondria-targeting design strategy, renew and expansion of clinical applicability on the conventional mitochondria-targeting drug is another potential translation

## Conclusion and prospects

Accumulating evidence has highlighted the importance of mitochondria homeostasis in maintaining immune cell differentiation and activation (33). However, alterations in mitochondrial functions, such as excessive mROS and mtDNA production, impaired mitophagy, altered mitochondrial dynamics, and disrupted redox homeostasis, initiate an immune cascade preceding the onset and progression of SLE (127). Critically, mitophagy downregulation results in the release of autoimmunogenic mtDNA that promotes the production of inflammatory cytokines, such as IL-1β and IFN-α (46). Targeting on mROS scavengers and mitophagy agonists are advantageous in SLE clinical treatment (185). In this review, mitophagy defects of antioxidant defense and mitochondrial dysfunction in SLE were mainly discussed. Nevertheless, certain questions and limitations remain to be addressed. During metabolic processes, the TCA cycle occurs in the mitochondria and involves in not only energy supplement glucose and lipid metabolism pathways. As the sophisticated complexity of mitochondria function, the effect of TCA cycle on immunity is worth to be discussed elsewhere. Moreover, the potential pre-clinical mitochondria-targeting chemicals have been only briefly summarized according to the limited space.

Mitochondria-targeting are promising therapeutics due to the clinical benefits. However, several challenges remain to be addressed. One of these is an intensively debated mechanism involved in mitochondria directing the cell fate in SLE (186). During the pathogenic conditions of systemic and chronic SLE, mitophagy and mitochondrial metabolism pathways form extremely complex networks that mutually affect immune signaling activation (7). Specific mROS scavenger and mitophagy agonist/antagonist are required for their function assessment in cellular fate decision and deepening the understanding of the mitochondria-associated immunometabolism (184). With the aid of the latest single-cell, multi-omics, and visualization technologies, identification of key mitochondrial metabolites that determine cell fate may shed light on SLE pathogenesis.

Furthermore, interdisciplinary research in pharmaceutics and materials engineering is required to develop a novel drug-targeting delivery platform that portrays great potential for SLE treatment, facilitating the release of drug payload in an organelle-specific and controllable manner for manipulating the cell differentiation (152). In addition, drug repurposing, which involves investigation of existing drugs for new therapeutic purposes, particularly uses biophysics-based molecular docking approaches and has been proven to be effective and efficient and of immense value in biomedicine (184, 187). The effect of repurposed drugs, such as metformin and CoQ10 in SLE treatment has been well investigated (75, 171). Therefore, our accumulated data help elucidate the mitochondrial metabolite landscape and in identifying mitochondria-targeting therapeutic strategies that precisely modulate abnormal cell differentiation in SLE. Nonetheless, the safety and efficacy of these strategies still require further investigation.

## Author contributions

All authors were involved in drafting the article or revising for important intellectual content, and all authors approved the final version to be published. LDZ and MW designed the literature search and wrote the article with input from all authors. LKZ, XH and MW drafted the manuscript. FX, LDZ and XZ revised the manuscript.

## Funding

This work was supported by grants from the National Natural Science Foundation of China (81788101, 82171798, 81801635, 81803848, and 32141004), the CAMS Innovation Fund for Medical Sciences (CIFMS) (2021-I2M-1-040,2021-I2M-1-017,2021-I2M-1-047,2021-I2M-1-016 and 2021-I2M-1-026).

## Conflict of interest

The authors declare that the research was conducted in the absence of any commercial or financial relationships that could be construed as a potential conflict of interest.

The reviewer JX declared a shared parent affiliation with the authors LKZ, FX, XZ, LDZ and MW to the handling editor at the time of review.

## Publisher’s Note

All claims expressed in this article are solely those of the authors and do not necessarily represent those of their affiliated organizations, or those of the publisher, the editors and the reviewers. Any product that may be evaluated in this article, or claim that may be made by its manufacturer, is not guaranteed or endorsed by the publisher.

## Glossary

**Table:**

3-PEHPC	3-pyridinyl ethylidene hydroxyl phosphonocarboxylate
8-OHdG	8-hydroxy-2-deoxyguanosine
AIM2	absent in melanoma 2
AMPK	AMP-activated protein kinase
ATP	adenosine triphosphate
BNIP3	BCL2 interacting protein 3
cGAS	cyclic GMP-AMP synthase
CoQ10	coenzyme Q10
DAMPs	damage-associated molecular patterns
Drp1	dynamin-related protein
ETC	electron transport chain
F/B	Firmicutes/Bacteroidetes
FAO	fatty acid oxidation
Fc&gamma; R	Fc&gamma; chain receptor
FUNDC1	FUN14 domain-containing protein 1
GPX	glutathione peroxidase
GPX4	glutathione peroxidase-4
H2S	hydrogen sulfide
HIF-1&alpha;	hypoxia-inducible factor 1 subunit alpha
IEC	intestinal epithelial cells
IFN	interferon
IFN-&alpha;	type I interferon
IL	interleukin
LN	Lupus Nephritis
Mfn1/2	mitofusin 1/2
MitoQ	mitoquinone
mROS	mitochondrial reactive oxygen species
mROS	mitochondrial ROS
mtDNA	mitochondrial DNA
mTOR	mammalian target of rapamycin
mTORC1	mammalian target of rapamycin complex 1
NAC	N-acetylcysteine
NETs	neutrophil extracellular traps
NFAT	nuclear factor of activated T cell
NLRP3	NLR family pyrin domain-containing 3
NLRs	NOD-like receptors
NOD	nucleotide-binding oligomerization domain
NRF	nuclear respiratory factor
Opa1	optic atrophy protein 1
Ox-mtDNA	oxidative mitochondrial DNA
OXPHOS	oxidative phosphorylation
pDCs	plasmacytoid dendritic cells
PGC-1&alpha;	PPAR-&gamma; coactivator 1 alpha
PRC	PGC-1&ndash; related coactivator
PINK1	PTEN-induced kinase 1
PPAR	peroxisome proliferator-activated receptor
RLRs	retinoic acid-inducible protein I (RIG-I)-like receptors
ROS	reactive oxygen species
SCFAs	short-chain fatty acids
SLE	systemic lupus erythematosus
SOD2	superoxide dismutase
STING	stimulator of interferon genes
TCA	tricarboxylic acid
TCR	T cell receptor
TFAM	transcription factor A
Th17	T helper 17
TLR	Toll-like receptors
Tregs	regulatory T
ULK1	unc-51 like autophagy-activating kinase 1
VDAC	voltage-dependent anion-selective channel.
